# Physical rehabilitation for people with advanced dementia who fracture their hip – expert consensus process

**DOI:** 10.1080/09638288.2023.2260739

**Published:** 2023-09-21

**Authors:** Abigail J. Hall, Fay Manning, Victoria A. Goodwin

**Affiliations:** a Public Health and Sports Science Department, University of Exeter, Exeter, UK; b Department of Medical Imaging, University of Exeter, Exeter, UK

**Keywords:** Dementia, hip fracture, rehabilitation, advanced

## Abstract

**Purpose:**

Hip fracture is common in older people – with prevalence even higher for people with dementia. Research often excludes people with dementia – especially those in the more advanced stages. Therefore, the most appropriate interventions remain unknown. The main aim of this study was to gain consensus about the core considerations needed to deliver a physical intervention for people with advanced dementia who fracture their hip. Materials and Methods: An expert consensus process was undertaken, using Nominal Group Technique, to explore the key considerations when delivering rehabilitation. Data collection was undertaken in January 2023 and involved an online group discussion followed by voting and off-line rating. Qualitative content analysis and quantitative analysis of consensus scoring was undertaken. An international group of seven highly specialised physiotherapists took part.

**Results:**

59 statements were agreed following the process. Content analysis was used to categorise these statements according to the International Classification of Functioning, Disability and Health. Although consensus levels were high, there was disagreement in several areas.

**Conclusion:**

The statements provide an overarching understanding of the multidisciplinary expertise that is needed to effectively deliver rehabilitation interventions to this population. People with dementia require highly skilled and trained professionals, providing holistic and person-centred approaches to deliver rehabilitation interventions.

**IMPLICATIONS FOR REHABILITATION**
- The expert consensus provides an overarching understanding of the multidisciplinary expertise that is needed to effectively deliver rehabilitation interventions to this population.
- Physiotherapy - or other interventions - cannot be used in isolation.
- People with dementia require highly skilled and trained professionals, providing holistic and person-centred approaches to deliver rehabilitation interventions.
- While our focus was on hip fracture, we suggest these statements can be used for people with advanced dementia with a variety of other conditions.

## Introduction

Hip fracture is a common and serious injury, especially prevalent in older people, with an estimated 65,000 such fractures occurring every year in the UK. It results in the occupation of over 4000 inpatient beds at any time in the UK [1], as such, hip fracture represents a significant economic burden to the UK each year.

People who fracture their hip often have co-morbidities [2], of which it is estimated that dementia is the most dominant, with people with dementia being 2.7 times more likely to fracture their hip than sex and age matched controls without dementia [3]. Dementia is an umbrella term used to describe a set of disorders that affect the brain, with over 100 established different types [4], but it can be broadly categorised into four main types: Alzheimer’s, vascular, Lewy Body and Frontotemporal – although many have mixed aetiologies. Despite having different symptoms and trajectories of disease progression, dementia results in a global and continuing loss of cognitive and intellectual functioning, leading to difficulty maintaining social and occupational performance [5].

As a chronic and progressive disease, it is ultimately a fatal neurodegenerative condition [6]. The initial stages of dementia may only present with discrete and almost undetectable symptoms, however, advanced dementia is characterized by profound cognitive impairment, absence of verbal communication and complete functional dependence [6]. In combination with the detrimental effects of a hip fracture, patients with advanced dementia represent a challenging population to treat. There is little evidence about how best to provide rehabilitation for people with advanced dementia [7–9], with studies often excluding people with dementia [10], especially those with advanced dementia [7,11], despite them representing a significant proportion of people who experience hip fracture. This means that research findings, including those for rehabilitation, may not be generalisable to the entire population of people with hip fracture. To ensure that this is not the case, research studies should be more inclusive [12–14]. It is suggested that interventions for people with dementia require a greater emphasis on adopting a biopsychosocial model, where functioning and participation is key [15]. However, to date, there is no evidence to inform clinicians what this entails.

### Aim

The main aim of this study was to gain consensus about the core considerations needed to deliver a physical rehabilitation intervention for people with advanced dementia who fracture their hip.

## Materials and methods

### Design

To explore the components of a physical rehabilitation intervention for people with advanced dementia following hip fracture, an expert consensus process was undertaken following the principles of Nominal Group Technique (NGT) [16]. The meeting was facilitated by AH and FG (both post-doctoral researchers in health sciences with a robust qualitative publication history and extensive training courses) and took place online using Microsoft Teams. The meeting was recorded and lasted approximately 90 min. NGT is one of the most common approaches to gaining consensus and incorporates an Action Research principle during a focus group. The study was reported using COREQ (see supplementary material) to ensure clear and transparent methods

### Participants

This study required experts within the field of physical rehabilitation, so a purposive sampling strategy was employed to recruit participants to take part in the expert consensus process. Participant inclusion required the physiotherapists to be experts in dementia care with advanced clinical skills and experience. It was decided to recruit physiotherapist to the study as there is currently little evidence to support interventions that physiotherapists deliver. It was anticipated that the interventions suggested may span wider than the domain of physiotherapy. Emails were sent to the potential participant. If they didn’t respond within 10 working days, a follow up email was sent. Pre-existing networks were drawn upon to recruit participants with the specialised skills that were required. Members of the patient and public involvement (PPI) group were asked to be part of the panel to ensure that the wider context was considered from the patient and public perspective. Some of the participants were known to the researcher from previous research, as such they were aware of the wider programme of work that the researchers were undertaking.

### Consent

The potential participants were provided with a participant information leaflet and were asked to provide written informed consent. Participants were informed that they were under no obligation to participate in the expert consensus process, and they could withdraw from it at any time, without any negative consequence.

### Service user involvement

A patient and public involvement (PPI) group was developed to help inform the research study. This group has been evolving and developing and now consists of people with a variety of experiences and interests in dementia, including those living with dementia, carers of people with dementia and healthcare professionals with personal experience of dementia.

Representatives of the PPI group took part in the expert consensus process. One member joined the meeting whereby they contributed fully to the discussion and then had an opportunity to discuss the statements before they were sent out to the panel for voting. This panel member contributed to the voting and the scores were added to the other participants on an equal basis. Further members of the PPI group who were not able to attend the focus group were also consulted about the statements at a later date. One of the major points raised was around the terminology used and several statements were re-worded to ensure clarity of understanding.

### Design

The expert consensus process followed an initial phase of data collection from focus groups exploring health care professionals treating people with advanced dementia. The expert consensus sought to refine this data and in conjunction with other evidence, determine key considerations when providing physical rehabilitation interventions to this population.

NGT was chosen as a method used within a participatory action research paradigm. This approach emphasizes the importance of the participant being an “active ingredient” in the research itself, rather than just being passive participants as occurs when undertaking a survey. It also allows the participant to use their own knowledge of their experiences, and thus the expertise of research participants is included in the research design, analysis, and results output.

All participants were provided with an information booklet (supplementary material) which detailed the NGT process, had summaries of evidence that currently exists for this population as well as the questions that were asked during the consensus process alongside the key domains for people to consider.

A nominal group technique (NGT) was used consisting of:

**Stage 1. Introduction**

An online presentation was provided to panel members, detailing the virtual NGT process. It explained the context in which to base ideas and the NGT questions for deliberation. It also provided the opportunity for any questions or clarifications.

**Stage 2. Silent idea generation**

Panel members were provided with approximately 10 min per question (during which participant microphones and video functions were turned off) to consider their ideas. Facilitators (AH and FM) were available *via* the platform’s chat function to answer any questions.

To help organise the ideas generated, a list of domains was displayed on screen. Domains were used to help organise responses during the next stage of the process. These domains were determined from our previous qualitative work [17]. It was made clear to participants that these domains were simply ideas to help organise their thoughts, but should not restrict their thinking.

Participants were asked the same initial question - ‘From an intervention perspective, what treatments/techniques should be implemented to effectively rehabilitate people with advanced dementia following hip fracture when they have the following current physical abilities;

- No/poor sitting balance
- Able to transfer by standing with equipment
- Transfer and able to mobilise with/without aid independently

This question was chosen as a prompt to allow the participant to contribute their ideas about what (if any) interventions or treatments were appropriate. These physical abilities were chosen as guides to consider and represented the extremes of ability that a person may have following hip fracture. The time since fracture was not specified which allowed participants to suggest core components of interventions that would be relevant at all stages of rehabilitation. The setting of the intervention was also not specified, again to give the experts the opportunity to consider core elements that would be relevant regardless of location. We purposively sampled our participants to be expert physiotherapists from a wide variety of settings to ensure various settings and contexts were considered. Exemplar domains were given to participants to consider, these were developed as a result of our previous qualitative work [17] and included:

- Approach
- Building rapport
- Reducing barriers
- Positioning
- Transfers/mobility
- Pain
- Sensory considerations
- Communication
- Assessment
- Environment
- Team knowledge/experience
- Outcome measures
- Time
- Involving others

**Stage 3. Round Robin**

Participants were asked to enable their video and microphones. A member of the research team (AH) facilitated each panel member to offer a single idea, in turn, in response to the question. This process continued until all the ideas were exhausted. All ideas were typed by a second researcher (FM) on to a live document, based on the domains shared previously, visible to all members in real-time, through screen sharing.

**Stage 4. Clarification stage**

Each domain was explored and any unclear ideas discussed to ensure accurate understanding. The meeting concluded when all panel members were satisfied that there were no outstanding queries.

**Stage 5. Individual scoring**

Prior to sharing with the panel members, the previously live document was formatted, converting ideas into single statements for participants to rate. An online questionnaire was then sent to the participant. In line with previous research, a 9-point Likert scale was used for panel members to vote on the statements generated – these range from not important/do not agree (1) to important/strongly agree (9). Once the agreed statements were generated, a second round of voting allowed participants to clarify if a statement was relevant to more than one of the scenarios. These were rated either as “yes” (to include) or “no” (not to include). 75% was set as the level of consensus required to include the statement for that particular scenario.

### Analysis

#### Qualitative analysis

Qualitative analysis was undertaken prior to the rating of statements. This was a pragmatic decision due to the number of statements and the duplication or similarity of statements. The research team are experienced qualitative researchers with a background in health services research. All have undertaken appropriate and extensive training in qualitative research methods and have a robust publication history using qualitative methodologies.

A process of content analysis [18] was undertaken. This first required the familiarisation of the statements for each of the scenarios. Any duplicate statements were removed at this stage, with discussion amongst the two researchers (AH + FM) to ensure that the meaning of the statement was not lost or misunderstood. Primary coding of the statements was undertaken by the primary researcher (AH) and then each statement was categorised into wider parent categories which was discussed with the second researcher (FM). A process of inductive and deductive coding followed, whereby the statements were organised according to the overarching meaning. Iterative coding was then undertaken to re-organise and restructure the statements and align with the International Classification of Functioning, Disability and Health (ICF) [19]. All statements were included and categorised for rating during the voting stage. Once analysed into key categories, the data were presented back to participants to check it was a true reflection of their thoughts. PPI representatives were asked to comment on the statements. The terminology was changed for several statements to reduce ambiguity, but no changes were made that altered the meaning of the statement. Three statements which had multiple components were separated to allow participants to rate each component rather than a single statement.

#### Quantitative analysis

To determine consensus the scoring responses from the online survey were exported to Microsoft Excel. The level of agreement was set at 75% of all participants within the set ranges, 1–3 (not important), 4–6 (equivocal) and 7–9 (important). In the case of strong disagreement (which we considered to be when one panel member scored 1 and another scoring 9), the outlier would be removed. Simple descriptive statistics were used for each individual statement which reached consensus, to highlight dispersion. Statements with over 75% consensus as being “important” were included. A second round of voting was then undertaken to clarify if the statement related to one or more of the scenarios.

## Results

Seven experts took part in the consensus process as well as PPI representatives (Table 1). All the experts were physiotherapists with an average of 21 years of clinical experience and 18 years working with people with dementia. All those approached agreed to participate and nobody dropped out during the study.

**Table 1.** Participant characteristics.

Sex	Female	7
	Male	0
Years of clinical experience	0–10	1
	11–15	1
	16–20	1
	21–25	1
	26–30	1
	30+	2
Years of experience working with people with dementia	0–9	2
	**10–15**	2
	16–20	0
	21–25	3
Location	England	5
	Scotland	1
	Denmark	1
Setting of work	In patient	2
	Strategic	1
	In patient	1
	Community	4
	Other	1

A total of 153 statements were generated across the three scenarios, with 125 (82%) statements achieving consensus of over 75% agreement and 70 (46%) achieving 100% consensus, representing a high level of agreement. The mean score was 8.2/9 suggesting high levels of importance for the statements (Table 2).

**Table 2.** Consensus levels for each scenario.

	100% consensus		> 75 % consensus		< 75% consensus	
	Number	Percentage	Number	Percentage	Number	Percentage
Little or no sitting balance	24	44	45	83	9	17
Able to transfer	19	42	37	82	8	18
Able to mobilise with/without aid	27	49	43	78	13	24
**Overall**	**70**	**45**	**125**	**81**	**30**	**19**

Content analysis of the statements resulted in them being categorised into the five ICF categories – body structure and function, activities, participation, environmental factors and personal factors. Initial second order codes were categorised and organised into broader categories (first order) and then further refined according to the ICF categories (Table 3).

**Table 3.** Categories developed during content analysis.

First Order (ICF Classification)	2nd Order	3rd Order
Body structure and function	General health	Hydration
	Co-morbidities	Continence
	Limitations	Exercises
		Cognition
		Pain
Participation	Goals	Functional activities
	Functional ability	Maintenance of abilities
		Ensure suitable clothing
		Promote quality of life
		Wheelchair assessments
		Engaging with family
Activities	Enjoyable activities	Washing and dressing
	Activities of daily living	Toileting
		Eating and drinking
		Hobbies
Environmental	Equipment	Seating provision
	Others present	Mobility equipment
		Availability of food and drinks
		Reduce restraint
		Carers
		Positive risk taking
		Family involvement
Personal factors	Consent	Building rapport
	Relationship building	Life goals
	Personal preferences	Hearing and vision
		Psychological issues
		Clothing
		Footwear
		Relaxation
		Manage expectations
		Safety
		Communication

**Figure 1.**
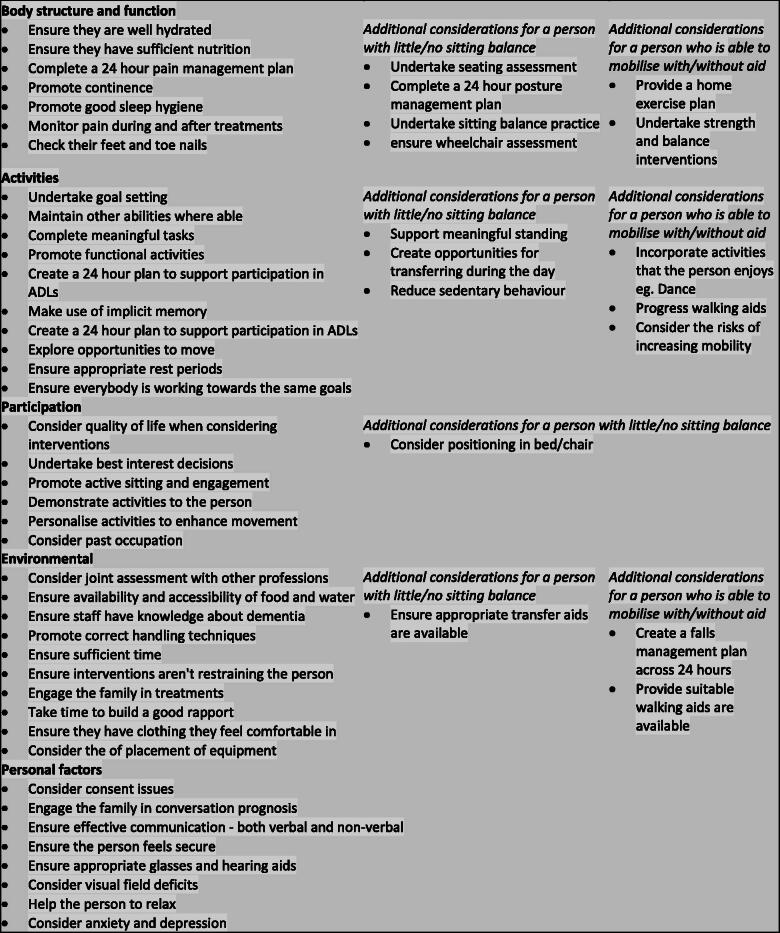
Included statements.

The first order categories related to the five ICF classifications, with a further twelve second order and 31 third order classifications. There was considerable overlap between scenarios and also between the ICF classifications, so duplicates were removed after the second round of voting, leaving a total of 59 statements (Figure 1).

### Areas of discordance

Although consensus levels were high, there was disagreement in several areas. These related to risk taking and discussion of end-of-life considerations.

Risk taking was considered to be a challenge and although many of our participants reported the importance of taking positive risk, it was questioned by all of our PPI representatives. As family members, they reported that any activity that increased the risk of injury or harm to their loved one was very counter-intuitive and caused a significant amount of distress. What constituted “positive risk” was debated with a particular concern about encouraging increased activity levels – even when this increased the risk of falls and injury. This debate was balanced by discussion around ensuring interventions weren’t restraining the person.

While end of life discussions were deemed important for people who had little or no sitting balance, they were not considered important for somebody who was now mobile post fracture.

## Discussion

The main objective of this study was to gain consensus about the core considerations needed to deliver a rehabilitation intervention for people with advanced dementia who fracture their hip. We recruited physiotherapists who were highly specialist in treating this population and determined 43 core considerations that are required to provide effective interventions to people with dementia following hip fracture. We also determined seven additional considerations for people who may be able to mobilise following the hip fracture and a further nine for people that had little or no sitting balance following fracture. These categories were chosen to replicate the extremes of physical abilities that a person may have following hip fracture rather than having a specific focus on mobility. Interestingly, despite the expert consensus panel consisting of physiotherapists, very few of the included statements related to components that were specific to “traditional” physiotherapy techniques. Indeed, most statements related to the conditions that were needed to facilitate activity and participation. This supports our previous work, whereby physiotherapists have suggested that physiotherapy interventions may not be effective for this population [7,17,20].

Little rehabilitation research has focused on people with advanced dementia [11,17] and although we considered interventions through the lens of a person following hip fracture, the overarching principles could be used to guide interventions for people with advanced dementia with other conditions. The considerations generated highlight the complexity of this patient population and the importance of ensuring the person has the optimum environment and conditions to have potential to improve. “Rehabilitation potential” is a term that is often used to depict the ability that a person has to improve with the provision of rehabilitation [21] and was first recognised as a term in the 1950s [22]. People with advanced dementia are often considered not to have “rehab potential.” Our data suggests that there are extensive considerations needed to create the optimum conditions to enhance a person’s rehabilitation potential.

Many of the considerations determined from this study highlight the need for a comprehensive and joined up multi-disciplinary team approach to rehabilitation. Issues relating to nutrition and hydration were reported to be key as well as ensuring pain is managed as well as possible. Evidence suggests that nutrition can be improved and malnutrition reversed in people with very advanced dementia [23] and while pain is a complex and multifaceted challenge for people with dementia [24], often stemming from a lack of confidence of healthcare professionals to adequately assess pain levels [25], there is a suggestion that correct assessment and management of pain can reduce agitation, fatigue and confusion [8]. Such barriers as malnutrition and pain can disguise the ability of a person with dementia and our data suggests the key importance of addressing such issues before attempting any physical rehabilitation.

For people with dementia who can walk following a hip fracture, there was much debate about the importance of understanding “risk” and how promoting and encouraging mobility could increase this risk. The challenge of balancing risk of falls versus promoting mobility has been discussed in the acute setting [26], where falls sensors are commonplace. However, there appears little by way of guidance as to when attempting mobility may constitute a risk that is too great. Our PPI representatives reported that as a family member of somebody with dementia, there was a real desire to “keep them safe” and a fear that promoting mobility was against their wishes. There is a growing body of literature exploring the conflicting perspectives of family carers, healthcare professionals and the person with dementia [27,28]. The challenge appears to be balancing physical risk, such as falls, with the emotional risk of restricting movement [29].

## Strengths and limitations

This study used a structured process of NGT with a small, but expert, group of physiotherapists. The use of the structured approach adds reliability to the results of the study. While we recognise that the size of the expert panel was small, they were purposively sampled due to their expertise. Physiotherapists with such expertise are rare, therefore there was a limited availability of people who were able to join the process. The consensus process resulted in high levels of agreement with statements, with only a small number of statements being excluded for lacking consensus. We do recognise that this may have excluded some statements which represented new or novel techniques that are not commonplace. We also chose 75% as a level of consensus based on a pragmatic decision due to the lack of supporting evidence to guide the process.

## Conclusion

This is the first study to undertake an expert consensus process to determine the considerations needed to provide rehabilitation for people with advanced dementia. Although we used hip fracture as a lens to focus our exploration, the key statements can be considered relevant to many other conditions and comorbidities for people with advanced dementia. Interventions designed for this population should seek to ensure that these considerations are incorporated into their design to optimise conditions for the person with advanced dementia to have the greatest opportunity for meaningful rehabilitation. The value of multi-disciplinary approaches to this population were key and clinical interventions must consider the wide range of professionals who are needed to support such patients.

## Supplementary Material

Supplemental Material

## Abbreviations

ICF: International Classification of Functioning, Disability and Health
NGT: Nominal Group Technique
PPI: Patient and Public Involvement

## References

[1] Royal College of Physicians. National hip fracture database (NHFD) annual report. 2015.

[2] de Luise C, Brimacombe M, Pedersen L, et al. Comorbidity and mortality following hip fracture: a population-based cohort study. Aging Clin Exp Res. 2008;20(5):412–418. 19039282 10.1007/BF03325146

[3] Melton LJ, Beard CM, Kokmen E, et al. Fracture risk in patients with alzheimer’s disease. J Am Geriatr Soc. 1994;42(6):614–619. 8201146 10.1111/j.1532-5415.1994.tb06859.x

[4] Draper B. Understanding alzheimer’s disease and other dementias. London, UK: Jessica Kingsley Publishers; 2013.

[5] McGilton KS, Davis AM, Naglie G, et al. Evaluation of patient-centered rehabilitation model targeting older persons with a hip fracture, including those with cognitive impairment. BMC Geriatr. 2013;13(1):136. 24330470 10.1186/1471-2318-13-136 PMC4028934

[6] Murphy E, Froggatt K, Connolly S, et al. Palliative care interventions in advanced dementia. Cochr Database System Rev. 2016;9(9):CD011513. 10.1002/14651858.CD011513.pub2 PMC6463843 27911489

[7] Hall AJ, Febrey S, Goodwin VA. Physical interventions for people with more advanced dementia–a scoping review. BMC Geriatr. 2021;21(1):675. 34863094 10.1186/s12877-021-02577-0 PMC8642899

[8] Ries N, Mansfield E, Sanson-Fisher R. Planning ahead for dementia research participation: insights from a survey of older australians and implications for ethics, law and practice. J Bioeth Inq. 2019;16(3):415–429. 31297689 10.1007/s11673-019-09929-x

[9] West E, Stuckelberger A, Pautex S, et al. Operationalising ethical challenges in dementia research—a systematic review of current evidence. Age Ageing. 2017;46(4):678–687. 28104596 10.1093/ageing/afw250

[10] Taylor JS, DeMers SM, Vig EK, et al. The disappearing subject: exclusion of people with cognitive impairment and dementia from geriatrics research. J Am Geriatr Soc. 2012;60(3):413–419. 22288835 10.1111/j.1532-5415.2011.03847.x

[11] Hall AJ, Fullam J, Lang IA, et al. Community physiotherapy for people with dementia following hip fracture: fact or ­fiction? A qualitative study. Dementia (London). 2020;19(8):2750–2760. 31219697 10.1177/1471301219857027

[12] Goodwin VA, Low MSA, Quinn TJ, et al. Including older people in health and social care research: best practice recommendations based on the INCLUDE framework. Age Ageing. 2023;52(6):afad082. 37261448 10.1093/ageing/afad082 PMC10234283

[13] Shepherd V, Hood K, Wood F. Unpacking the ‘black box of horrendousness’: a qualitative exploration of the barriers and facilitators to conducting trials involving adults lacking capacity to consent. Trials. 2022;23(1):471. 35668460 10.1186/s13063-022-06422-6 PMC9167903

[14] Shepherd V, Wood F, Griffith R, et al. Protection by exclusion? The (lack of) inclusion of adults who lack capacity to consent to research in clinical trials in the UK. Trials. 2019;20(1):474. 31382999 10.1186/s13063-019-3603-1 PMC6683336

[15] Spector A, Orrell M. Using a biopsychosocial model of dementia as a tool to guide clinical practice. Int Psychogeriatr. 2010;22(6):957–965. 20561384 10.1017/S1041610210000840

[16] Delbecq AL, Van de Ven AH, Gustafson DH. Group techniques for program planning: a guide to nominal group and Delphi processes. Scott: Foresman; 1975.

[17] Hall AJ, Manning F, Goodwin V. Key considerations when providing physical rehabilitation for people with advanced dementia. Int J Environ Res Public Health. 2023;20(5):4197. 36901207 10.3390/ijerph20054197 PMC10001442

[18] Krippendorff K. Content analysis: an introduction to its methodology. London, UK: Sage publications; 2018.

[19] World Health Organization. IFC: international classification of functioning, disability and health. Geneva, Switzerland: WHO; 2001.

[20] Hall AJ, Manning F, Goodwin V. Qualitative study exploring health care professionals’ perceptions of providing rehabilitation for people with advanced dementia. BMJ Open. 2023;13(7):e072432. 10.1136/bmjopen-2023-072432 PMC10391829 37524545

[21] Cowley A, Goldberg SE, Gordon AL, et al. Rehabilitation potential in older people living with frailty: a systematic mapping review. BMC Geriatr. 2021;21(1):533. 34620112 10.1186/s12877-021-02498-y PMC8496021

[22] Whiting HS. Classification of rehabilitation potential. J Rehabil. 1950;16(6):7–9. 14795499

[23] Biernacki C, Ward L, Barratt J. Improving the nutritional status of people with dementia. Br J Nurs. 2001;10(17):1104–1114. 11904569 10.12968/bjon.2001.10.17.9949

[24] Fry M, Arendts G, Chenoweth L, et al. Cognitive impairment is a risk factor for delayed analgesia in older people with long bone fracture: a multicenter exploratory study. Int Psychogeriatr. 2014;27(2):1–6. 25162158 10.1017/S1041610214001732

[25] Jonsdottir T, Gunnarsson EC. Understanding nurses’ knowledge and attitudes toward pain assessment in dementia: a literature review. Pain Manag Nurs. 2021;22(3):281–292. 33334680 10.1016/j.pmn.2020.11.002

[26] Growdon ME, Shorr RI, Inouye SK. The tension between promoting mobility and preventing falls in the hospital. JAMA Intern Med. 2017;177(6):759–760. 28437517 10.1001/jamainternmed.2017.0840 PMC5500203

[27] Cott CA, Tierney MC. Acceptable and unacceptable risk: balancing everyday risk by family members of older cognitively impaired adults who live alone. Health Risk Soci. 2013;15(5):402–415.

[28] Clarke CL, Gibb CE, Keady J, et al. Risk management dilemmas in dementia care: an organizational survey in three UK countries. Int J Older People Nurs. 2009;4(2):89–96. 20925808 10.1111/j.1748-3743.2008.00149.x

[29] Stevenson M, Taylor BJ. Risk communication in dementia care: family perspectives. J Risk Res. 2018;21(6):692–709.

