# Supplementary material for: Physical rehabilitation for people with advanced dementia who fracture their hip – expert consensus process
Source: Disabil Rehabil. 2023 Sep 21;46(17):3985–91. doi: 10.1080/09638288.2023.2260739 (PMC11332403; doi:10.1080/09638288.2023.2260739)
Supplement: Supplemental Material [file IDRE_A_2260739_SM2041.zip › info_booklet.docx]

**EXPERT CONSENSUS PROCESS**

**Nominal Group Technique**

**Information Booklet**

**November 2022**

Contents

[Background to Study 3](#_Toc120532130)

[Purpose and context 3](#_Toc120532131)

[Target population 3](#_Toc120532132)

[Subgroups 3](#_Toc120532133)

[Target setting 3](#_Toc120532134)

[Nominal Group Technique 5](#_Toc120532135)

[Questions for the Nominal Group Technique Process 6](#_Toc120532136)

[Patient and carer experience 7](#_Toc120532137)

[Summary of existing evidence 8](#_Toc120532138)

[References 10](#_Toc120532139)

# Background to Study

Hip fracture is a common injury, especially in older people, with over 70,000 people sustaining a hip fracture in the UK each year, costing £2 billion in health and social care [1]. People who fracture their hip often have multiple co-morbidities of which it is estimated that dementia is the most prevalent, with studies reporting that 19 to 40% of older adults with a hip fracture have dementia [2].

People with dementia who fracture their hip have more complex care needs with greater risk of complications, physical disabilities and social care requirements compared with people without dementia [3]. Indeed, the outcomes for people with dementia following hip fracture are poor, with a two-fold increase in mortality at twelve months post-operatively [4] and a higher risk of morbidity [5]. This population experience longer hospital stays and a significantly greater proportion will require long term care home placement [6], with only 30% of people with dementia returning home within 30 days of fracture [6].

Several systematic reviews suggest the benefits of exercise in people with dementia to improve/maintain physical function [7], balance [8] and fitness [9]. Other reviews of physical interventions have suggested improved cognitive function [10] as well as improvements in levels of depression and behavioural difficulties [11]. However, the majority of these studies only include participants with mild to moderate dementia, and there is a paucity of evidence regarding physical rehabilitation interventions for people with more advanced dementia [12]. Therefore the aim of this project is to refine and develop a complex intervention designed to support physiotherapists working in primary care settings to manage this population, which is recognised as an under-served group [13].

# Purpose and context

The purpose of the expert consensus process is to generate a set of treatment components which can be used as a “toolkit” for therapists when treating people with advanced dementia following hip fracture. The intervention will include physical rehabilitation techniques which are tailored to the needs of the person depending on their current abilities.

## Target population

The target population is people with advanced dementia who have fractured their hip. Advanced dementia is characterised by profound cognitive impairment, potential absence of verbal communication and functional dependence. Thus, they would be rated 2-3 on the Clinical Dementia Rating Scale (Figure 1).

## Subgroups

The interventions will be discussed in relation to people of varying physical abilities.

1. No/poor sitting balance
2. Able to transfer by standing with equipment
3. Transfer and mobile independently

## Target setting

The intervention is designed to be a community based intervention – whether this is in a residential or nursing home, or in the person’s own home. Thus, any intervention needs to be feasible to deliver in such settings.


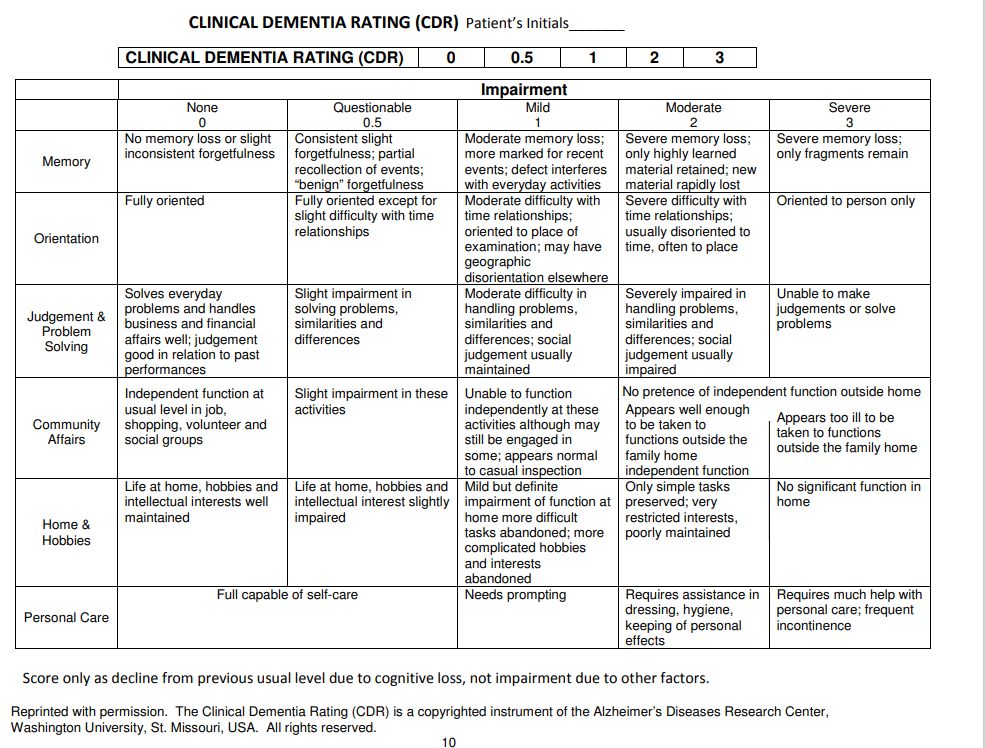


Figure 1 - adapted Clinical Dementia Rating (CDR) Scale

# Nominal Group Technique

Nominal (meaning in name only) group technique (NGT) is a structured variation of a small-group discussion to reach consensus. NGT gathers information by asking individuals to respond to questions posed by a moderator, and then asking participants to prioritize the ideas or suggestions of all group members.

**1, Introduction**

Online presentation shared – explanation of process, re-iterate aims

Opportunity for questions

**2, Silent idea generation**

Video and sound turned off for 10 minutes. Participants generate ideas

**3, Round robin**

Each participant presents one idea at a time. This continues until all ideas are exhausted. The process is repeated for each question.

**4, Clarification**

Ideas are discussed to ensure full understanding

Meeting concludes when there are no further questions or comments

**5, Scoring**

Ideas are created into statements. Statements sent out to participants in a survey. Rating scale (0-9) used to rate importance of ideas.

Answers are then correlated and analysed for consensus

# Questions for the Nominal Group Technique Process

During the meeting, you will be asked to consider the following question. There are domains which you may wish to consider, but please note that these are just guides to start generating ideas. It is perfectly acceptable if your ideas do not relate to any of these. We will consider the question for each of the different levels of physical abilities.

***‘From an intervention perspective, what treatments / techniques should be implemented to effectively rehabilitate people with advanced dementia following hip fracture when they have the following current physical abilities;***

1. ***No/poor sitting balance***
2. ***Able to transfer by standing with equipment***
3. ***Transfer and mobile independently’***

Domains to consider;

- Approach
- Building rapport
- Reducing barriers
- Positioning
- Transfers/mobility
- Pain
- Sensory considerations
- Communication
- Assessment
- Environment
- Team knowledge/experience
- Outcome measures
- Time
- Involving others

# Patient and carer experience

As part of the overall project, we have a group of people (who may be patients) and carers who are involved in the development of the project and ensure that we are considering the patient context in all aspects of our work. As part of the initial discussions with this group of people, a word cloud (Figure 2) was generated to summarise some of their key thoughts.


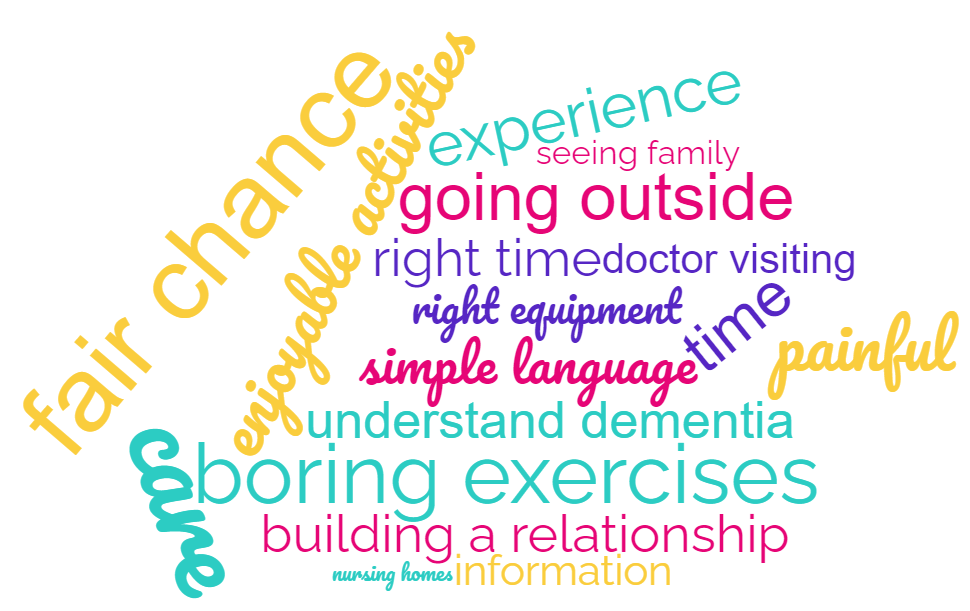


Figure 2 - word cloud generated from discussions with patients and their carers

# Summary of existing evidence

**NICE guidance**

The following NICE guidance is relevant to this population and may help to inform this study.

**Pain for people living with dementia**

1.8.3 Consider using a structured observational pain assessment tool:

- alongside self-reported pain and standard clinical assessment for people living with moderate to severe dementia
- alongside standard clinical assessment for people living with dementia who are unable to self-report pain.

1.8.4 For people living with dementia who are in pain, consider using a stepwise treatment protocol that balances pain management and potential adverse events.

1.8.5 Repeat pain assessments for people living with dementia:

- who seem to be in pain
- who show signs of behavioural changes that may be caused by pain
- after any pain management intervention.

**Multidisciplinary management of hip fracture (not dementia specific)**

1.8.6 Patients admitted from care or nursing homes should not be excluded from rehabilitation programmes in the community or hospital, or as part of an early supported discharge programme.

The following table (Table 1) demonstrates the summary of data from a scoping review we undertook [14] exploring randomised controlled trials for people with advanced dementia – this study was not specific to people with hip fracture as the review failed to find any studies specifically for people with advanced dementia following hip fracture.

Table 1 - summary of randomised controlled trials included in scoping review [14]

| **Study details** |  |  |  |  |
| --- | --- | --- | --- | --- |
| Lead author | **Burge** | **Francese** | **Kim** | **Venturelli** |
| Year | **2017** | **1997** | **2016** | **2011** |
| Country | Switzerland | USA | Korea | Italy |
| Sample size | 270 | 11 | 38 | 24 |
| Severity of dementia (mild, mod, advanced) | Moderate to severe | "Late stage" | Moderate to severe | Moderate to severe |
| Measurement of physical ability | Barthel, FIM | Physical therapy assessment, Tinetti | Exercise time, the number of pedal rotation, total load, grip strength, and the Berg Balance Scale | 6 minute walking test, Barthel Index |
| Intervention | Usual care + general exercise programme | Physical exercise programme | Physical exercise programme plus multicomponent cognitive programme | Walking |
| Who delivered | Physical therapists, occupational therapists, or "psychomotor therapists" | Primary investigator plus volunteer(s) | Physical therapist | Physiotherapist and caregiver |
| Method of delivery | Groups of 4 | Small groups | Not reported | Individual |
| Location of intervention | Acute psychiatric ward patients, but delivered off the ward | Dementia nursing facility | long term care facility | Alzheimer’s care unit |
| When/how much | 20 physical exercise sessions over 4 weeks lasting 30 mins each of moderate intensity | 20 minutes 3x week for 7 weeks | 60 min of supervised exercise sessions 5 times a week for 6 months | 30minutes of moderate exercise (walking) 4 times a week over 24 weeks |
| Results | ADL scores deteriorated; the exercise program delays the loss of mobility but does not have a significant impact on overall ADL scores. | Significant improvements in Tinetti measurement with the intervention | Exercise time, Berg balance score and grip strength were significantly increased at 6 months | Walking and ADLs improved in the intervention group |

#

# References

1. National Institute for Health and Clinical Excellence, *Hip fracture in adultes: NICE quality standard*. 2016, NICE: UK.

2. Seitz, D.P., et al., *Prevalence of dementia and cognitive impairment among older adults with hip fractures.* Journal of the American Medical Directors Association, 2011. **12**(8): p. 556-64.

3. Smith, T.O., et al., *Enhanced rehabilitation and care models for adults with dementia following hip fracture surgery.* Cochrane Database of Systematic Reviews, 2015(6).

4. Ruggiero, C., et al., *Early post-surgical cognitive dysfunction is a risk factor for mortality among hip fracture hospitalized older persons.* Osteoporosis International, 2017. **28**(2): p. 667-675.

5. Berggren, M., et al., *Co-morbidities, complications and causes of death among people with femoral neck fracture–a three-year follow-up study.* BMC geriatrics, 2016. **16**(1): p. 120.

6. Royal College of Physicians, *National Hip Fracture Database (NHFD) annual report 2015*. 2015: UK.

7. Pitkälä, K., et al., *Efficacy of physical exercise intervention on mobility and physical functioning in older people with dementia: a systematic review.* Experimental gerontology, 2013. **48**(1): p. 85-93.

8. Suttanon, P., et al., *Can balance exercise programmes improve balance and related physical performance measures in people with dementia? A systematic review.* European Review of Aging and Physical Activity, 2010. **7**(1): p. 13-25.

9. Heyn, P., B.C. Abreu, and K.J. Ottenbacher, *The effects of exercise training on elderly persons with cognitive impairment and dementia: a meta-analysis.* Archives of physical medicine and rehabilitation, 2004. **85**(10): p. 1694-1704.

10. Hess, N.C., et al., *The effect of exercise intervention on cognitive performance in persons at risk of, or with, dementia: A systematic review and meta-analysis.* Healthy Aging Research, 2014. **3**(3).

11. de Souto Barreto, P., et al., *Exercise training for managing behavioral and psychological symptoms in people with dementia: A systematic review and meta-analysis.* Ageing Research Reviews, 2015.

12. Hall, A.J., S. Febrey, and V.A. Goodwin, *Physical interventions for people with advanced dementia – a scoping review to explore the effects on physical outcomes.* BMC Geriatrics (in press), 2021.

13. NIHR. *Improving inclusion of under-served groups in clinical research: Guidance from the NIHR INCLUDE project*. (2020) [cited 2021 29/9/2021]; Available from: [www.nihr.ac.uk/documents/improving-inclusion-of-under-served-groups-in-clinical-research-guidance-from-include-project/25435](https://universityofexeteruk-my.sharepoint.com/personal/a_hall4_exeter_ac_uk/Documents/REDEFINED/Expert%20consensus/Ethics/www.nihr.ac.uk/documents/improving-inclusion-of-under-served-groups-in-clinical-research-guidance-from-include-project/25435).

14. Hall, A.J., S. Febrey, and V.A. Goodwin, *Physical interventions for people with more advanced dementia–a scoping review.* BMC geriatrics, 2021. **21**(1): p. 1-10.
